# Potential lives saved in 73 countries by adopting multi‐cohort vaccination of 9–14‐year‐old girls against human papillomavirus

**DOI:** 10.1002/ijc.31321

**Published:** 2018-03-01

**Authors:** Mark Jit, Marc Brisson

**Affiliations:** ^1^ London School of Hygiene & Tropical Medicine London WC1E 7HT United Kingdom; ^2^ Public Health England London WC1E 7HT United Kingdom; ^3^ Centre de recherche du CHU de Québec, Université Laval London WC1E 7HT United Kingdom

**Keywords:** human papillomavirus, vaccination, low‐ and middle‐income countries, mathematical modeling

## Abstract

Up to 2016, low‐ and middle‐income countries mostly introduced routine human papillomavirus (HPV) vaccination for just a single age‐cohort of girls each year. However, high‐income countries have reported large reductions in HPV prevalence following “catch‐up” vaccination of multiple age‐cohorts in the year of HPV vaccine introduction. We used the mathematical model PRIME to project the incremental impact of vaccinating 10‐ to 14‐year‐old girls compared to routine HPV vaccination only in the same year that routine vaccination is expected to be introduced for 9‐year‐old girls across 73 low‐ and lower‐middle‐income countries. Adding multiple age‐cohort vaccination could increase the number of cervical cancer deaths averted by vaccine introductions in 2015–2030 by 30–40% or an additional 1.23–1.79 million over the lifetime of the vaccinated cohorts. The number of girls needed to vaccinate to prevent one death is 101 in the most pessimistic scenario, which is only slightly greater than that for routine vaccination of 9‐year‐old girls (87). These results hold even when assuming that girls who have sexually debuted do not benefit from vaccination. Results suggest that multiple age‐cohort vaccination of 9‐ to 14‐year‐old girls could accelerate HPV vaccine impact and be cost‐effective.

## Abstract 2

**What's new?**

To prevent cervical cancer, many low‐ and middle‐income countries employ HPV vaccination programs for girls of a certain age, usually 9. What about girls too old when the program starts? Here, the authors ask how many lives could be saved by offering vaccination at a wider age range. Using data from 73 countries, they determined that a vaccinating girls between ages 9 and 14 years could prevent 30–40% more cancer deaths, with a similar cost‐effectiveness. Routine vaccination of 9‐year‐olds prevents 1 death per 87 vaccinations; adding 10‐ to −14‐year‐olds only bumps that slightly to 101 vaccinations per death prevented.

**Abbreviations**
DoV: Decade of Vaccines
HPV: human papillomavirus
LMICs: low‐ and middle‐income countries
WHO: World Health Organization

Human papillomavirus (HPV) vaccination protects vaccinees against HPV infection, a necessary cause of cervical cancer. Cervical cancer kills 266,000 women every year, with 82% of them in low‐ and middle‐income countries (LMICs).^1^ Yet up to October 2014, only 1% of the 118 million females vaccinated against HPV were from LMICs.^2^

HPV vaccine introduction in most high‐income countries was accompanied with multiple age‐cohort (multi‐cohort) or “catch‐up” vaccination during which females older than the age of routine vaccination were offered vaccination, with an upper limit of around 15–26 years depending on the country.^2^ These countries have already seen large reductions in HPV prevalence and (for countries using the quadrivalent vaccine) anogenital warts incidence across a wide age range.^3^ Rapid reduction in anogenital warts incidence following HPV vaccination in countries like Australia has been largely attributed to catch‐up vaccination.^4^

In contrast, LMICs have mostly introduced routine HPV vaccination for just a single age‐cohort of girls each year. Until 2016, the World Health Organization (WHO) recommended prioritizing routine vaccination of 9‐ to 13‐year‐old girls without any mention of multi‐cohort vaccination.^5^ Funding for vaccination from Gavi, the Vaccine Alliance, only covered vaccination of a single age‐cohort per year.

In October 2016, the WHO's Strategic Advisory Group of Experts on Immunization revised its position to recommend delivering vaccination to multiple age‐cohorts of girls aged 9–14 years.^6^ Shortly afterward, Gavi opened a funding envelope to allow countries to vaccinate multiple age‐cohorts of girls aged 9–14 years in the year of HPV vaccine introduction.^7^ Expanding multi‐cohort vaccination to LMICs offers the opportunity to accelerate the slow pace of HPV vaccination in these settings. However, there are concerns that the impact of multi‐cohort vaccination will be curtailed if girls are likely to be infected with vaccine‐type HPV prior to being vaccinated, as HPV vaccines do not affect prevalent infections at the time of vaccination.

To address these concerns, we conducted data analysis and modeling work to project the potential incremental impact of multi‐cohort vaccination in 73 Decade of Vaccines (DoV) countries projected to introduce HPV vaccination in 2015–2030. DoV countries are those that the Global Vaccine Action Plan for 2011–2020 focuses on consisting of countries classified as low or lower‐middle income by the World Bank in 2011.^8^ These analyses were used to inform the decisions by WHO and Gavi to support multi‐cohort vaccination.

## Methods

### Model overview

We estimated the impact of different HPV vaccination strategies in DoV countries using the Papillomavirus Rapid Interface for Modelling and Economics (PRIME). PRIME is a static, proportional impact model of HPV vaccination that was developed in collaboration with WHO to estimate the impact and cost‐effectiveness of introducing HPV vaccination in LMICs. It is also used to inform vaccine impact estimates used by Gavi and the Bill & Melinda Gates Foundation.

The model equations and inputs have been extensively described elsewhere^9^ and the Excel‐based code with accompanying documentation is freely available online (http://www.primetool.org). Herd (indirect) effects and cross‐protection against nonvaccine HPV types are not considered, so impact estimates for routine vaccination should be regarded as conservative. However, previous validation exercises suggest that PRIME gives comparable cost‐effectiveness estimates for routine female‐only vaccination to transmission dynamic models in the literature.^9^ As PRIME was originally designed to measure the impact of vaccinating girls prior to sexual debut, for this exercise, we adjusted the original model to take into account that vaccinating up to age 14 may involve giving vaccines to some girls who are already HPV infected (see below for details).

### Vaccine effectiveness

We assume that vaccinating girls prior to infection with HPV types 16 and 18 fully protects them from developing cervical cancer caused by HPV 16 and 18, in accordance with vaccine trials.^10^ However, the proportion of girls aged 9–14 years who are HPV 16/18 infected is not available for most DoV countries. As a proxy for HPV infection, we examined sexual behavior in the same age group. We explored two scenarios: a pessimistic scenario in which vaccination would give no protection at all to girls who had sexually debuted, and an optimistic scenario in which vaccination would still fully protect these girls.

### Data sources

Data sources for model parameters are summarized in Supporting Information, Appendix 4. Of 94 DoV countries, we excluded 15 not projected to introduce HPV vaccination in 2015–2030 and 6 lacking both sexual activity and World Development Indicator information. For the remaining 73 countries (Supporting Information, Appendix 3), input parameters used in previous publications^9^ were used apart from the exceptions below:

1. Country population. United Nations World Population Prospects 2015 figures were obtained for number of females in the 5–9 and 10–14‐year‐old age groups in 2015–2030.
2. Vaccine coverage. HPV vaccine introduction years and subsequent vaccine coverage were based on Gavi's Strategic Demand Forecast version 12, released in 2015.^11^ This forecast represents Gavi's best estimates about countries' expected time of introduction and corresponding vaccine coverage, but does not constitute any commitment or obligation by the countries. Many countries are forecast to have an initial period of coverage scale‐up, with low vaccine coverage in the year of introduction that gradually increases to a higher projected level of coverage several years after vaccine introduction. Vaccine coverage in the year of introduction ranges from 6% to 79% (average by country 63%) and rises to reach a maximum of 50–99% (average by country 91%) (Table 1). We also considered a low‐coverage scenario in which vaccine coverage of the relevant age‐cohort never exceeds 45%, the coverage of ≥2 doses among United States females in 2015–2016.^12^
  **Table 1:** HPV vaccine introduction years and subsequent vaccine coverage for each WHO region, based on Gavi's Strategic Demand Forecast version 12

		Year of vaccine introduction		Eventual coverage achieved (%)	
WHO region	Countries	Fastest country	Slowest country	Lowest country	Highest country
AFR	39	2011	2028	61	99
AMR	10	2013	2023	80	99
EMR	11	2017	2028	50	98
EUR	12	2017	2023	94	99
SEAR	9	2016	2025	89	99
WPR	15	2009	2022	78	99
All	96	2009	2028	50	99
3. Age at sexual debut. Demographic and Health Survey (DHS) data report the proportion of females who have become sexually active by age 15, 18, 21 and 25 years. DHS data were available for 53 out of 73 countries comprising 84% of the 9‐year‐old female age‐cohort (Supporting Information, Appendix 3).

### Extrapolation of sexual activity data

Data are not available for sexual activity before age 15 years. Hence, we fitted two functions (a logit function and a gamma cumulative distribution function) to data at the four ages with data, giving equal weight to each point, that is, we chose the values of *a* and *b* to minimize the sum of squared residuals between the proportion of sexually active females at age *x* years and the function *f*(*x*) = 1/(1 + *e* ^‐^^*a*^^(^^*x*^^+^^*b*^^)^) (logit) or *f*(*x*) = 1/Γ(*a*) γ(*a*,*bx*) (gamma). The best fitting of the two models (based on the deviance) was used to extrapolate sexual debut in females younger than 15 years.

We validated our model using sexual debut data in 12‐ to 30‐year olds from Benin,^13^ three cities in Kartanaka, India (Mysore, Bellary and Belgaum),^14^ the United States,^15^ Canada^16^ and the United Kingdom.^17^ We fit the gamma model to data at age 15, 18, 20 and 25 years only, and examined whether the model was able to reproduce data at the other ages well.

Of the 73 countries, we examined, 20 had no relevant DHS sexual activity data. These were matched to countries with such data in a three‐step process based on similarity of other variables: (i) using linear regression to select predictors of female sexual activity at age 15 years from a basket of indicators in the 53 countries with data, (ii) using an clustering algorithm to partition 73 countries into eight clusters based on similarities in the predictors of sexual activity and (iii) matching countries without relevant data to the same‐cluster country with the highest proportion of sexually active females at age 15 years. Technical details of these procedures are given in Supporting Information, Appendix 2.

### Vaccine strategies and outcomes

We compared two scenarios: (i) the current Gavi scenario, in which only 9‐year‐old girls are offered vaccination and (ii) a multi‐cohort vaccination scenario, in which girls aged 9–14 years are offered vaccination in the first year of vaccine introduction. In subsequent years, only 9‐year‐old girls are vaccinated. We assumed that a multi‐cohort campaign would enable first year coverage in all catch‐up age groups to be equal to the highest routine coverage attained. An alternative scenario at 75% of the highest routine coverage was also explored. Vaccinations expected to take place in the period 2015–2030 were considered.

The primary outcome is the number of deaths due to cervical cancer prevented by vaccinating these cohorts over the lifetime of the vaccinated cohorts. Results are presented aggregated over (i) the year in which vaccination is delivered and (ii) the year in which the outcome (averted deaths) occurred.

As a secondary outcome, we calculated the number needed to vaccinate to prevent one cervical cancer‐related death, a common metric used to describe the efficiency of HPV and other vaccines.^18^ This was defined as the ratio of the number of fully vaccinated girls divided by cervical cancer deaths prevented over the lifetime of the vaccinated girls.

## Results

**Figure 1:**
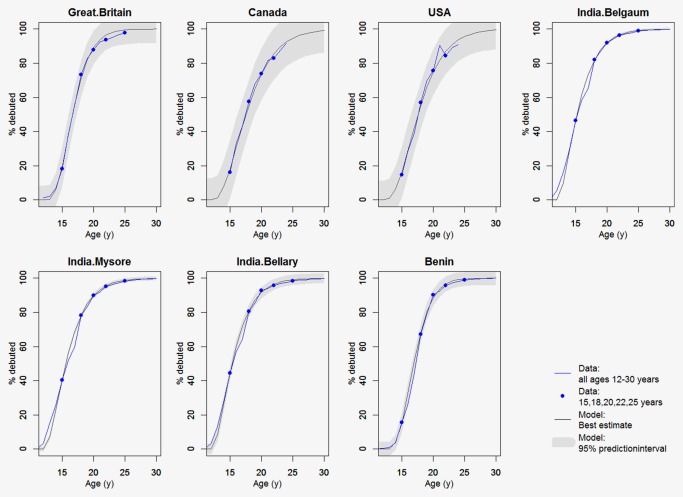
Sexual activity data from seven settings and corresponding best fitting gamma model when fit to data at age 15, 18, 20, 22 and 25 years only. Prediction intervals are generated using Monte Carlo sampling from the variance–covariance matrix of the regression coefficients. [Color figure can be viewed at http://wileyonlinelibrary.com]

The proportion of girls reported in DHS to be sexually active at age 15 ranges from 0.3% (Turkmenistan, Ukraine) to 35.0% (Chad), with a mean proportion of 14.4% averaged over countries. The gamma model fit DHS data on sexual activity better than a logit model, with deviance of 0.15 (gamma) versus 0.46 (logit) (Supplemental Appendix 1). The gamma model was also able to reproduce sexual activity data on 12‐ to 30‐year olds in Benin, India, the United States, Canada and the United Kingdom (Fig. 1).

**Figure 2:**
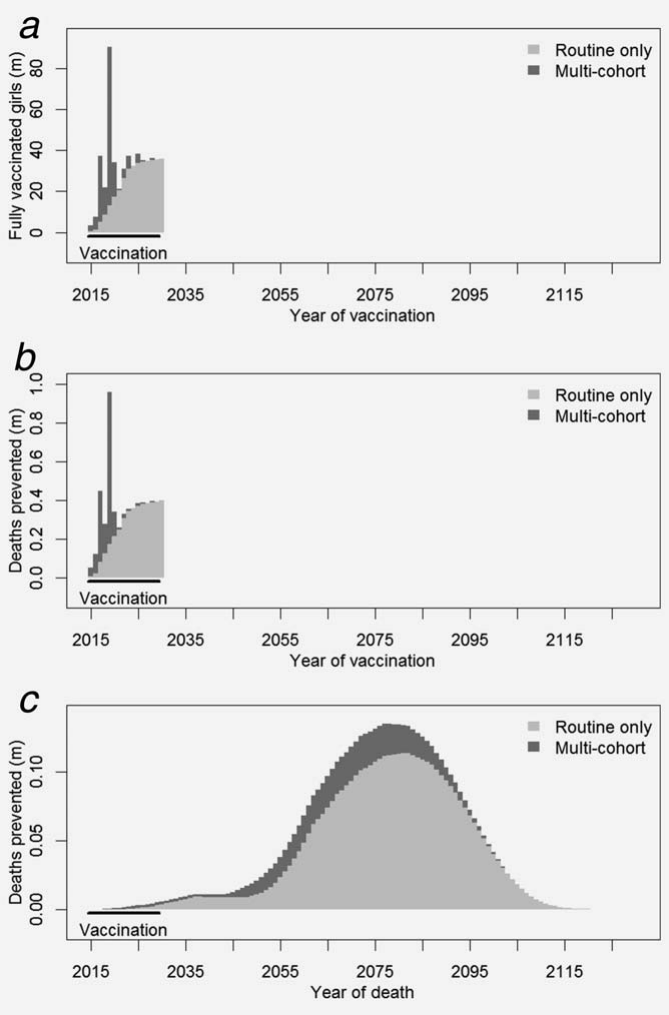
Number of (*a*) fully vaccinated girls, (*b*) cancer deaths prevented by year of vaccination and (*c*) cancer deaths prevented by year that death occurs, when vaccinating over the SDF v. 12 time period (2015–2030) with routine vaccination only compared to multi‐cohort vaccination at 100% routine coverage.

Multi‐cohort vaccination accelerates the impact of HPV vaccination on cervical cancer. If routine vaccine introductions in 2015–2030 are accompanied with multi‐cohort vaccination for 10‐ to 14‐year‐old girls in the same year, then an additional 1.23–1.79 million deaths would be averted over the lifetime of the vaccinated cohorts (Table 2 and Fig. 2). This represents a 30–40% proportional increase in deaths prevented compared to vaccinating 9‐year‐old girls alone. This assumes that sexually active vaccines are not protected; the uncertainty range represents differences between achieving 75% and 100% of maximum routine coverage. Multi‐cohort vaccination also accelerates the health benefits from vaccination, as can be seen in Figure 2 *c* where the deaths averted from vaccinating 10‐ to 14‐year‐old girls occur earlier than deaths averted from vaccinating 9‐year routine cohorts. The number of 10‐ to 14‐year‐old girls that need to be vaccinated to prevent one cancer death in multi‐cohort vaccination is 101, compared to 87 for routine vaccination at 9 years. If a more optimistic scenario is assumed where sexually active girls are still protected by vaccination, then the number needed to vaccinate to prevent one cancer death in multi‐cohort vaccination is 93. The results of the two scenarios are similar, indicating that there is limited sexual activity below age 15.

**Table 2:** Number of fully vaccinated girls and lives saved by vaccination during 2015–2030 under different coverage and vaccine protection scenarios explored

	Total			Incremental to routine only		
Scenario	Fully vaccinated girls (m)	Lives saved (m)	Number needed to vaccinate	Fully vaccinated girls (m)	Lives saved (m)	Number needed to vaccinate
Routine at 9 years	366	4.2	87	‐	‐	‐
Pessimistic scenario: sexually active vaccines are not protected						
+ Catch‐up 9–14 years at 100% of routine coverage	532	5.8	91	166	1.65	101
+ Catch‐up 9–14 years at 75% of routine coverage	491	5.4	91	124	1.23	101
Optimistic scenario: sexually active vaccines are protected						
+ Catch‐up 9–14 years at 100% of routine coverage	532	6.0	89	166	1.79	93
+ Catch‐up 9–14 years at 75% of routine coverage	491	5.5	89	124	1.34	93

Vaccination at age 10–14 is almost as efficient as vaccination at age 9. If single‐cohort HPV vaccination was introduced at the timing predicted by Gavi to females aged 9, 10, 11, 12, 13 or 14 years, then the number needed to vaccinate to prevent one cervical cancer death would be 87, 87, 87, 90, 98 or 111, respectively.

In the low‐coverage scenario, the number of lives incrementally saved by multi‐cohort vaccination of 10‐ to 14‐year olds halves to 0.63–0.91 million, but the number needed to vaccinate to prevent a death is largely unchanged (Supporting Information, Appendix 5).

## Discussion

Our results show that multi‐cohort vaccination of 10‐ to 14‐year‐old girls when routine HPV vaccination for 9‐year‐old girls is introduced could substantially increase the impact of vaccination by accelerating reduction in cervical cancer deaths. Up to 2016, the focus in DoV countries has been on delivering HPV vaccines to girls at the lower end of the age range for HPV vaccine indications (i.e., close to 9 years old). This is because vaccine effectiveness is reduced if vaccinees are HPV infected before vaccination.

The number of girls that need to be vaccinated to prevent a cancer death in multi‐cohort vaccination is only slightly greater than that for routine vaccination. As all females under 15 years are recommended to receive two doses of vaccine, if each 10‐ to 14‐year old can be given a vaccine dose at the same cost as a 9‐year old, then multi‐cohort vaccination would have a similar cost‐effectiveness profile as routine vaccination. As routine vaccination is cost‐effective in almost all countries in the world,^9^ there is a strong case that 10‐ to 14‐year‐old females should be entitled to the same benefits from vaccination as those aged 9 years.

This is the first paper to look at the impact of the new WHO recommendations on multi‐cohort vaccination in 10‐ to 14‐year‐old females in DoV countries. Most modeling papers looking at the impact of HPV catch‐up campaigns have been limited to high‐income countries.^19^, ^20^ Most such papers found catch‐up campaigns to be cost‐effective when compared to routine vaccination alone, although the upper age limit for cost‐effectiveness ranged between studies from 15 to 24 years. None of the 25 articles in a recent systematic review of HPV vaccine cost‐effectiveness studies in low‐ and middle‐income countries^21^ analyzed multi‐cohort vaccination. To our knowledge, only two papers (neither of them in the review) have looked at multi‐cohort HPV vaccination in LMICs. One paper^22^ found that catch‐up for 12‐ to 15‐year‐old girls in Poland and Guinea brought forward reductions in HPV infection by up to 5 years earlier, but did not look at the impact on cancer. A second paper^23^ found that very extensive catch‐up campaigns (11–26 and 11–75 years) increased the impact of female HPV vaccination in the Lao People's Democratic Republic.

Our analysis used PRIME, a static model that projects vaccine impact without requiring detailed information about sexual mixing and intermediate disease markers such as HPV prevalence or screening outcomes. It does not capture indirect (herd) effects on unvaccinated females as a result of reduced transmission in the population. However, the error in ignoring herd effects is small when evaluating vaccinating young females at coverage close to 100%. Given that 80/87 (92%) of the countries expected to introduce HPV vaccination between 2015 and 2030 are projected to achieve coverage of 80% or greater, the estimates using PRIME are likely to be satisfactory. Furthermore, transmission dynamic models have found that the benefits of vaccinating females in multi‐cohort campaigns are similar to vaccinating routine cohorts as long as the females are under around 15 years old. However, the precise magnitude of the herd effects depends on type‐specific transmission and clearance rates as well as the characteristics of the population in each country.

Coverage assumptions were based on Gavi projections of future vaccine demand. However, not many countries have achieved the high levels of coverage that Gavi projects. Furthermore, in several countries vaccine coverage has fallen following (unfounded) safety concerns.^24^ In a sensitivity analysis with coverage assumptions closer to those currently being achieved in the United States, the impact of multi‐cohort vaccination of 10‐ to 14‐year olds halves (Supporting Information, Appendix 5). Herd effects may be greater at lower levels of coverage, so this may partly mitigate the reduction. On the other hand, if 2 doses of HPV vaccines do not provide long‐lasting protection as we have assumed, then the impact of coverage declines will be greater. Extending vaccination to males may allow programs to achieve resilience against short‐term declines in coverage.^25^

Another simplification is that we assumed that any girl who has sexually debuted is HPV 16/18 infected and does not benefit from HPV vaccination at all. This assumption allows us to adjust the differences in HPV exposure using only data on the onset of sexual debut across the countries. However, the proportion of 15‐year olds who have sexually debuted does not exceed 35% in any DoV country with relevant DHS data, and is substantially lower in most countries. Hence even when making this extremely pessimistic assumption, the effectiveness of vaccination at age 14 is only slightly lower than at age 9. For comparison, although 18% of British females are sexually active at age 15,^17^ prior to widespread HPV vaccination, fewer than 5% were seropositive for HPV 16 or 18 at that age.^26^ Similarly, in the PATRICIA trials of the bivalent HPV vaccine, 96% of participants were sexually active, but only 19% and 13% were either antibody or DNA positive to HPV 16 and HPV 18, respectively.^27^

All these model simplifications lead to our analysis underestimating the benefit of vaccination, that is, the benefit of multi‐cohort vaccination may be even greater than we show here. Hence we have followed WHO guidelines, which allow the use of a conservative static model (that underestimates vaccine impact) provided that this still produces outcomes that are favorable to vaccination.^28^

A further limitation is that we assume (like most published models) that cervical cancer incidence will not change in the future. Future incidence depends on trends in sexual behavior, screening uptake, HIV prevalence, all‐cause mortality and other factors. However, long‐term cervical cancer incidence projections taking all relevant factors into account have never been published.

While the impact of multi‐cohort vaccination is potentially large, there are still delivery questions that need to be addressed. First, multi‐cohort vaccination will require much larger HPV vaccine stocks, particularly in 2018 when several large countries are predicted to introduce HPV vaccination. Second, female school enrolment in many countries drops after primary school. Hence school‐based vaccination may have lower coverage in multi‐cohort age groups compared to routine cohorts. Furthermore, DHS data indicate an association between being sexually active by age 15 and not having secondary or postsecondary education at the country level (data not shown). This may suggest that out‐of‐school girls are more likely to be at risk of HPV infection and disease. Hence vaccinating girls at secondary school age may require strategies that are able to reach out‐of‐school girls to have maximal impact.

## Supporting information

Supporting Information
