# Supplementary material for: Potential lives saved in 73 countries by adopting multi‐cohort vaccination of 9–14‐year‐old girls against human papillomavirus
Source: Int J Cancer. 2018 Mar 1;143(2):317–23. doi: 10.1002/ijc.31321 (PMC6001440; doi:10.1002/ijc.31321)
Supplement: Supplementary file 1 — Supporting Information [file IJC-143-317-s001.docx]

**Potential lives saved in 73 countries by adopting multi-cohort vaccination of 9-14 year old girls against human papillomavirus vaccination**

**Supplementary Appendix**

**Appendix 1.** Proportion of females who are sexually active at age 15, 18, 21 and 25 years in all countries with relevant DHS data (black dots), together with the best fitting logit function (black line) and its 95% prediction interval (shaded area). Prediction intervals are generated using Monte Carlo sampling from the variance-covariance matrix of the regression coefficients.


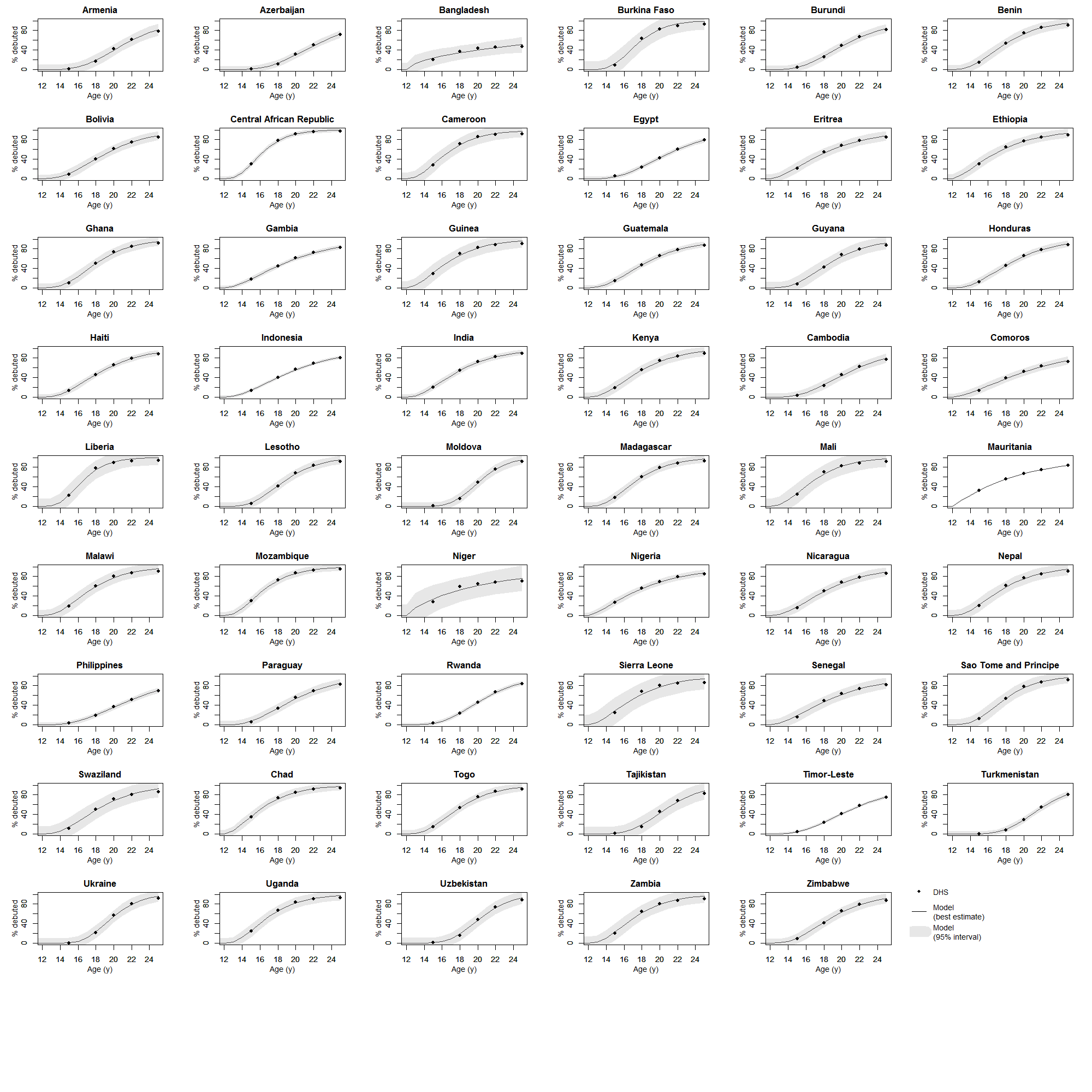


**Appendix 2.** Matching countries without DHS data on sexual activity to countries with these data.

Of the 73 countries we examined, 20 had no relevant DHS data on sexual activity. These were matched to countries with DHS data in the following steps:

***Step 1: Identifying country-level variables that predict sexual activity at age 15 years***

For the 53 countries with data on the proportion of 15-year old females who were sexually active, the following country-level data were retrieved from the World Development Indicators collection (World Bank; see <http://data.worldbank.org/products/wdi>):

- Adolescent fertility rate (births per 1,000 women ages 15-19), 2015
- Primary completion rate, female (% of relevant age group), 2010-2016
- GDP per capita, PPP (constant 2011 international $), 2011-2016
- Rural population (% of total population), 2011-2016
- Maternal mortality ratio (modeled estimate, per 100,000 live births), 2015
- School enrolment, primary and secondary (gross), gender parity index (GPI), 2010-2016
- Literacy rate, youth female (% of females ages 15-24), 2016
- Proportion of seats held by women in national parliaments (%), 2015-2016

For each country we used data for the latest year available (2016 in the majority of cases); the date range above shows the range across the 53 countries. These variables together with WHO region (as a categorical variable), were then used in a linear model as predictors of the proportion of females who were sexually active at age 15 years. The variables corresponding to the most parsimonious model (based on the Aikaike Information Criterion) were then selected using backward stepwise regression (using the R function *step*). The most parsimonious model is summarised in Table A1.

| **Indicator** | **Estimate** | **Standard Error** | **t-statistic** | **p** |
| --- | --- | --- | --- | --- |
| (Intercept) | 3.34×10^-1^ | 8.30×10^-2^ | 4.018 | 0.000296 |
| Adolescent fertility | 8.43×10^-4^ | 2.83×10^-4^ | 2.98 | 0.005214 |
| Female primary completion | -2.83×10^-3^ | 6.91×10^-4^ | -4.091 | 0.00024 |
| Rural population | -1.19×10^-3^ | 6.35×10^-4^ | -1.876 | 0.069002 |
| Maternal mortality | 6.73×10^-5^ | 4.96×10^-5^ | 1.356 | 0.183853 |
| WHO region: Americas | 3.31×10^-3^ | 3.32×10^-2^ | 0.1 | 0.921157 |
| WHO region: Eastern Mediterranean | 4.06×10^-2^ | 6.46×10^-2^ | 0.628 | 0.534111 |
| WHO region: European | -7.25×10^-3^ | 4.06×10^-2^ | -0.178 | 0.859547 |
| WHO region: South-East Asian | 1.40×10^-1^ | 3.94×10^-2^ | 3.567 | 0.00107 |
| WHO region: Western Pacific | -3.45×10^-3^ | 6.38×10^-2^ | -0.054 | 0.957152 |

Table A1. Regression coefficients, t-statistics and p-values for each term in the most parsimonious model for indicators of the proportion of females sexually active at age 15 years.

***Step 2: Matching countries based on indicators of female sexual activity at age 15 years***

The 73 countries were partitioned into clusters based on their distance from each other in terms of the indicators in the most parsimonious model in Step 1. The Partitioning Around Medoids (PAM) clustering algorithm^1^ was used with distances defined by the Gower Similarity Coefficient^2^ because of the mixture of continuous and categorical variables.

The number of clusters was varied between 2 and 20, with the optimal number of 8 chosen based on the Average Silhouette Width (ASW).^1^ The change in ASW with the number of clusters is shown in Figure A1. Figures A2 and A3 show details of each cluster; it can be seen that the optimal clusters are defined around WHO region except for the African region which is partitioned into three clusters defined mainly around adolescent fertility and maternal mortality.

All clustering computations were conducted using the R package *cluster* (functions *pam* and *daisy*).

***Step 3: Extrapolating sexual activity data to countries within the same cluster***

We matched each of the 20 countries without relevant DHS data to the country with the highest proportion of females who were sexually active at age 15 years in the same cluster. Choosing the country with the highest proportion ensured that our matching was conservative (i.e. that we were more likely to underestimate vaccine impact in older females). We then used the best fitting gamma function of sexual activity by age in that country in all the same-cluster countries without relevant data.


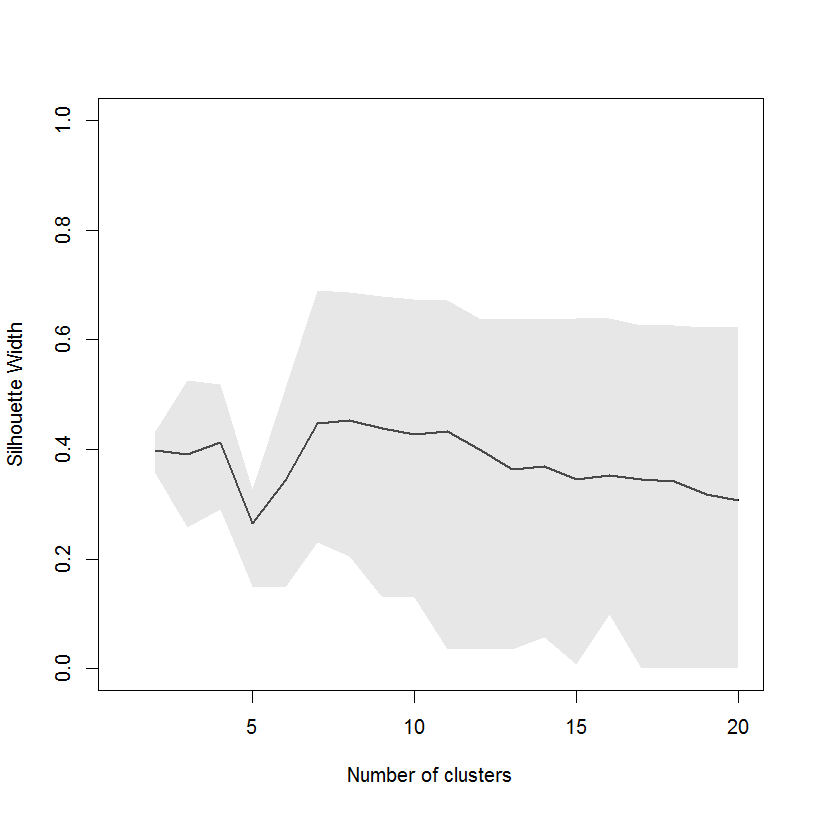


Figure A1. Silhouette Width of a partition with a given number of clusters. The line shows the average while the shaded region shows the range between the maximum and minimum.

******

Figure A2. Country-level indicators for countries in each of the eight clusters in the optimal partitioning.

**

Figure 3. Countries in each cluster (shown by their ISO-3 codes in the bottom right corner) and proportion of females who are sexually active by age for countries with relevant data. Colour of lines corresponds to colour of country names in the bottom right corner.

**Appendix 3.** Details for the 73 Decade of Vaccine (DoV) countries with results presented in this paper.

| Country | WHO region | Lives saved (routine only) | Lives saved (multi-cohort) | % additional lives saved by multi-cohort | Is DHS data available? | Country match if no DHS data |
| --- | --- | --- | --- | --- | --- | --- |
| Angola | AFR | 85300 | 109000 | 28% | No | Chad |
| Armenia | EUR | 952 | 1330 | 40% | Yes |  |
| Azerbaijan | EUR | 3230 | 4220 | 31% | Yes |  |
| Burundi | AFR | 66600 | 90900 | 36% | Yes |  |
| Benin | AFR | 25400 | 34300 | 35% | Yes |  |
| Burkina Faso | AFR | 46600 | 62700 | 35% | Yes |  |
| Bangladesh | SEAR | 136000 | 189000 | 39% | Yes |  |
| Belize | AMR | 829 | 1110 | 34% | No | Nicaragua |
| Bolivia, Plurinational State of | AMR | 34000 | 45800 | 35% | Yes |  |
| Bhutan | SEAR | 438 | 588 | 34% | No | India |
| Côte d'Ivoire | AFR | 40700 | 53700 | 32% | No | Chad |
| Cameroon | AFR | 45700 | 59400 | 30% | Yes |  |
| Congo, the Democratic Republic of the | AFR | 192000 | 267000 | 39% | No | Chad |
| Congo | AFR | 6800 | 9340 | 37% | No | Mauritania |
| Comoros | AFR | 3740 | 5190 | 39% | Yes |  |
| Cape Verde | AFR | 1130 | 1460 | 29% | No | Mauritania |
| Cuba | AMR | 4620 | 6320 | 37% | No | Nicaragua |
| Djibouti | EMR | 795 | 1170 | 47% | No | Egypt |
| Egypt | EMR | 9640 | 16100 | 67% | Yes |  |
| Eritrea | AFR | 6610 | 10000 | 51% | Yes |  |
| Ethiopia | AFR | 169000 | 225000 | 33% | Yes |  |
| Georgia | EUR | 1000 | 1640 | 64% | No | Uzbekistan |
| Ghana | AFR | 83700 | 110000 | 31% | Yes |  |
| Guinea | AFR | 28100 | 40900 | 46% | Yes |  |
| Gambia | AFR | 8960 | 11600 | 29% | Yes |  |
| Guinea-Bissau | AFR | 3690 | 5120 | 39% | No | Chad |
| Guatemala | AMR | 26400 | 34800 | 32% | Yes |  |
| Guyana | AMR | 1670 | 2190 | 31% | Yes |  |
| Honduras | AMR | 12900 | 17400 | 35% | Yes |  |
| Haiti | AMR | 9260 | 15200 | 64% | Yes |  |
| Indonesia | SEAR | 176000 | 245000 | 39% | Yes |  |
| India | SEAR | 961000 | 1480000 | 54% | Yes |  |
| Kenya | AFR | 127000 | 175000 | 38% | Yes |  |
| Kyrgyzstan | EUR | 6780 | 10100 | 49% | No | Uzbekistan |
| Cambodia | WPR | 23200 | 32800 | 41% | Yes |  |
| Lao People's Democratic Republic | WPR | 6110 | 8100 | 33% | No | Cambodia |
| Liberia | AFR | 10700 | 14500 | 36% | Yes |  |
| Sri Lanka | SEAR | 6900 | 10100 | 46% | No | India |
| Morocco | EMR | 35500 | 48000 | 35% | No | Egypt |
| Moldova, Republic of | EUR | 1920 | 2690 | 40% | Yes |  |
| Madagascar | AFR | 102000 | 132000 | 29% | Yes |  |
| Mali | AFR | 83600 | 111000 | 33% | Yes |  |
| Myanmar | SEAR | 51000 | 70000 | 37% | No | India |
| Mongolia | WPR | 3450 | 4630 | 34% | No | Cambodia |
| Mozambique | AFR | 158000 | 204000 | 29% | Yes |  |
| Mauritania | AFR | 9370 | 12600 | 34% | Yes |  |
| Malawi | AFR | 106000 | 136000 | 28% | Yes |  |
| Niger | AFR | 23500 | 29400 | 25% | Yes |  |
| Nigeria | AFR | 277000 | 389000 | 40% | Yes |  |
| Nicaragua | AMR | 13800 | 18900 | 37% | Yes |  |
| Nepal | SEAR | 33300 | 46100 | 38% | Yes |  |
| Pakistan | EMR | 75100 | 113000 | 50% | No | Egypt |
| Papua New Guinea | WPR | 14000 | 19800 | 41% | No | Cambodia |
| Sudan | EMR | 14100 | 25800 | 83% | No | Egypt |
| Senegal | AFR | 71100 | 92300 | 30% | Yes |  |
| Solomon Islands | WPR | 1500 | 2090 | 39% | No | Cambodia |
| Sierra Leone | AFR | 16400 | 22000 | 34% | Yes |  |
| El Salvador | AMR | 6490 | 9000 | 39% | No | Nicaragua |
| South Sudan | AFR | 8250 | 15900 | 93% | No | Ethiopia |
| Sao Tome and Principe | AFR | 655 | 929 | 42% | Yes |  |
| Chad | AFR | 4510 | 11600 | 157% | Yes |  |
| Togo | AFR | 15100 | 20200 | 34% | Yes |  |
| Tajikistan | EUR | 3810 | 5980 | 57% | Yes |  |
| Turkmenistan | EUR | 2480 | 3640 | 47% | Yes |  |
| Timor-Leste | SEAR | 1570 | 2280 | 45% | Yes |  |
| Tanzania, United Republic of | AFR | 306000 | 388000 | 27% | No | Mauritania |
| Uganda | AFR | 181000 | 225000 | 24% | Yes |  |
| Ukraine | EUR | 11800 | 17300 | 47% | Yes |  |
| Uzbekistan | EUR | 20800 | 28300 | 36% | Yes |  |
| Viet Nam | WPR | 28700 | 45300 | 58% | No | Cambodia |
| Yemen | EMR | 4340 | 8020 | 85% | No | Egypt |
| Zambia | AFR | 73300 | 94600 | 29% | Yes |  |
| Zimbabwe | AFR | 55800 | 74100 | 33% | Yes |  |

Excluded countries (21 countries):

*Introduced HPV vaccination before 2015 (8 countries):* Fiji, Micronesia, Kiribati, Lesotho, Marshall Islands, Paraguay, Rwanda, Vanuatu

*Not projected to introduce HPV vaccination by 2030 (7 countries)*: Central African Republic, Philippines, West Bank and Gaza, Swaziland, Syria, Tonga, Samoa

*Lacking one or more Wolrd Development Indicators (6 countries):* Afghanistan, Iraq, Korea (Democratic People’s Republic), Somalia, Tuvalu, Kosovo. Haiti and Turkmenistan also lack one or more World Development Indicators but have DHS data on sexual behaviour by age.

**Appendix 4.** Sources for parameters used in the model

| **Parameter** | **Source** |
| --- | --- |
| Vaccine efficacy | Assumed to be 100% against vaccine-type infection in girls prior to sexual debut |
| All-cause mortality | All-cause mortality from 2009 from life tables provided by the World Health Organization Global Health Observatory ^4^ |
| Size of vaccinated cohorts (9-14-year old girls) | United Nations World Population Prospects 2015 for the number of 9 year old girls ^3^, then all-cause mortality (as above) used to estimate size of cohorts for older ages (using the life table approach) |
| Vaccine coverage | Gavi’s Strategic Demand Forecast version 12, released in 2015 (see Table 2 of main text) |
| Age at sexual debut | Demographic and Health Survey (DHS) data for 53 of the 73 countries, extrapolation for the other countries ^5^ |
| Cervical cancer incidence and mortality | GLOBOCAN 2012 ^6^ |
| % of cervical cancer due to HPV 16/18 | Data by geographical region in a meta-analysis conducted by the International Agency for Research on Cancer ^7^ |

**Appendix 5.** Number of fully vaccinated girls and lives saved by vaccination during 2015 – 2030 under different coverage and vaccine protection scenarios explored, assuming that vaccine coverage can reach a maximum of 45% of the relevant cohort.

|  | **Total** | | | **Incremental to routine only** | | |
| --- | --- | --- | --- | --- | --- | --- |
| **Scenario** | **Fully vaccinated girls (m)** | **Lives saved (m)** | **Number needed to vaccinate** | **Fully vaccinated girls (m)** | **Lives saved (m)** | **Number needed to vaccinate** |
| Routine at 9y | 197 | 2.24 | 88 | - | - | - |
| Pessimistic scenario: sexually active vaccinees are not protected | | | | | | |
| + Catch-up 9-14y at 100% of routine coverage | 281 | 3.08 | 91 | 84 | 0.83 | 100 |
| + Catch-up 9-14y at 75% of routine coverage | 260 | 2.87 | 91 | 63 | 0.63 | 100 |
| Optimistic scenario: Sexually active vaccinees are protected | | | | | | |
| + Catch-up 9-14y at 100% of routine coverage | 281 | 3.15 | 89 | 84 | 0.91 | 93 |
| + Catch-up 9-14y at 75% of routine coverage | 260 | 2.92 | 89 | 63 | 0.70 | 93 |

References

1. Kaufman L, Rousseeuw PJ. Finding Groups in Data: An Introduction to Cluster Analysis (Wiley Series in Probability and Statistics). John Wiley & Sons, 1990.

2. Gower JC. A General Coefficient of Similarity and Some of Its Properties. *Biometrics* 1971;27:857.

3. United Nations Population Division of the Department of Economic and Social Affairs. World Population Prospects: The 2015 Revision [Internet]. 2016 [cited 2017 Sep 29];Available from: https://esa.un.org/unpd/wpp/publications/

4. World Health Organization. WHO Global Health Observatory. Available from: http://apps.who.int/ghodata/. Accessed on 15 October 2010. 2010;

5. USAID. The DHS Program: Demographic and Health Surveys [Internet]. 2017 [cited 2017 Sep 29];Available from: https://dhsprogram.com/

6. Ferlay J, Bray P, Pizani P, Parkin DM. GLOBOCAN 2002: Cancer incidence, mortality and prevalence worldwide. *IARC CancerBase No 5, Version 2.0 IARCPress, Lyon*2004;

7. Li N, Franceschi S, Howell-Jones R, Snijders PJF, Clifford GM. Human papillomavirus type distribution in 30,848 invasive cervical cancers worldwide: Variation by geographical region, histological type and year of publication. *Int J Cancer* 2011;128:927–35.
